# Identifying moderating factors during the preschool period in the development of borderline personality disorder: a prospective longitudinal analysis

**DOI:** 10.1186/s40479-022-00198-6

**Published:** 2022-09-15

**Authors:** Kiran Boone, Alecia C. Vogel, Rebecca Tillman, Amanda J. Wright, Deanna M. Barch, Joan L. Luby, Diana J. Whalen

**Affiliations:** 1 Department of Psychology, Washington University in St. Louis, St. Louis, MO USA; 2 Department of Psychiatry, Washington University School of Medicine in St. Louis, 4444 Forest Park, Suite 2100, St. Louis, MO 63108 USA

**Keywords:** Borderline personality disorder, Developmental psychopathology, Moderating factors, Resilience, Observational coding

## Abstract

**Background:**

Despite a growing literature detailing early childhood risk factors for borderline personality disorder (BPD), few studies have examined moderating factors that might mitigate or exacerbate the effects of those risk factors. The current study examined whether three preschool-age characteristics—impulsivity, emotional lability, and initiative-taking—moderated the relationship between known preschool-age risk factors and adolescent BPD symptoms.

**Methods:**

We performed multilevel modeling analyses in a sample (*n* = 151) from the Preschool Depression Study, a prospective longitudinal study with assessments from preschool through adolescence. Preschool risk factors included adverse childhood experiences, internalizing symptoms, and externalizing symptoms measured with parent clinical interviews. Preschool moderating factors were assessed via parent report and observational coding of temperament and behavior. The Borderline Personality Features Scale for Children measured BPD symptoms in adolescence.

**Results:**

We found that observed initiative-taking moderated the relationship between preschool internalizing symptoms and adolescent BPD symptoms (*b* = 0.57, *p* = .011) and moderated the relationship between preschool externalizing symptoms and adolescent BPD symptoms (*b* = 1.42, *p* = .013). Greater initiative-taking was associated with lower BPD risk for children with high internalizing or externalizing symptoms. Conversely, for children with low internalizing or externalizing symptoms, greater initiative-taking was associated with increased BPD risk.

**Conclusions:**

We identify a potential moderating factor in BPD development, offer novel targets for screening and intervention, and provide a framework for using early childhood observational assessments in BPD research. Our findings suggest the need for future research on early moderating factors in BPD development, which could inform early childhood interventions targeting those factors to mitigate the effects of potentially less malleable risk factors.

**Supplementary Information:**

The online version contains supplementary material available at 10.1186/s40479-022-00198-6.

## Background

Borderline personality disorder (BPD) is characterized by instability across multiple domains, including affect, relationships, and self-image, as well as impulsivity associated with frequent risk-taking and self-destructive behavior [1]. Because patients with BPD are often prone to experiencing intense, difficult-to-regulate negative emotions and tendencies toward impulsive behavior, rates of suicidal behavior among those with the disorder have been reported to be as high as 78% [2], rates of self-harm as high as 91% [3], and rates of substance abuse as high as 50% [1, 4]. Although treatment of BPD with psychotherapy has been shown to be fairly effective at reducing severe symptoms [5, 6], early intervention could significantly improve the quality of life of those who would otherwise go on to maintain this debilitating and often life-threatening disorder [7, 8].

One key component of BPD prevention is the identification of the most salient risk and protective factors for BPD that could be targeted by early intervention. As is the case with many disorders, early life adversity, parental psychopathology, parenting style, childhood trauma, IQ, childhood temperament, and childhood psychopathology have each been found to predict later BPD diagnosis [9]. More recent work has focused on identifying risk factors specific to BPD and on determining their developmental timing to inform the optimal timing of intervention strategies. Geselowitz et al. [10] found that adverse childhood experiences (ACEs), maternal support, internalizing symptoms, and externalizing symptoms during the preschool period predicted adolescent BPD symptoms and together accounted for 20% of the variance in adolescent BPD symptoms; preschool ACEs alone were found to account for 14.9% of the variance in adolescent BPD symptoms. These findings suggest that early childhood adversity and psychopathology confer substantial risk for developing BPD, consistent with Crowell and colleagues’ [11] extension of Linehan’s [12] biosocial model of BPD development, which posits that transactions between an invalidating (including high adversity) environment and a child’s predisposition toward emotional sensitivity and negative affectivity contribute to increased risk for BPD. It follows that BPD intervention efforts could benefit from screening for and addressing ACEs and manifestations of psychopathology as early as the preschool period.

Implementing interventions earlier in childhood may be critical given recent evidence about the timing of BPD onset. Historically, BPD diagnoses were applied only to adult patients, but recent work has supported the validity of diagnosing BPD in adolescents. Adolescent BPD has been found to be: (a) comparable to adult BPD in the strength of its associations with childhood risk factors, comorbid disorders, and suicidal behavior and ideations [13]; (b) similar in features to adult BPD; (c) stable into adulthood for a significant subset of patients [14]; and (d) associated with significantly impaired functioning, both concurrently and later on throughout adulthood [10, 15–17]. As a result, much of the recent BPD development literature has shifted to using adolescent BPD symptoms, rather than adult symptoms, as the outcome of interest [10, 18, 19].

Given that early childhood adversity and psychopathology increase risk for later BPD diagnosis, and that BPD symptoms can emerge as early as adolescence, further work is needed to identify mechanisms for mitigating BPD risk at the earliest developmental point before the disorder’s debilitating symptoms emerge. Despite its robust association with BPD, childhood adversity does not invariably lead to BPD development, as evidenced by recent work examining siblings who experienced similar levels of adversity but were later discordant for BPD [20, 21]. Retrospective, qualitative analyses have indicated that individual differences in coping ability, initiative-taking, sociability, and optimism may serve as protective factors moderating the association between childhood trauma and later BPD diagnosis [21]. Beyond this work, however, there is little research on moderating factors in BPD development. There is a clear need for identifying moderating factors that may illuminate more specific targets for BPD intervention, which could have substantial utility in cases where targeting childhood adversity and psychopathology is costly, slow, complex, or impossible.

The current study begins to address this gap in the literature by prospectively examining characteristics evident at preschool age that might moderate the relationship between early childhood risk factors for BPD and BPD symptoms in adolescence. We chose from factors that had either been identified as moderating factors in prior retrospective work (including coping ability, initiative-taking, sociability, and optimism) [21] or were relevant to BPD features as described by the criteria for BPD diagnosis in the Diagnostic and Statistical Manual of Mental Disorders (DSM-5) (including identity disturbance, interpersonal deficits, emotional lability, and impulsivity) [22]. From these, we selected moderating factors that might reasonably be targeted by interventions and for which we had both parent and observer reports. This resulted in three individual-level, temperament-based hypothesized moderating characteristics of interest: impulsivity, emotional lability, and initiative-taking in early childhood, each described in more detail below. In using this strategy, we aimed to extend the predominant models of BPD development [11, 12] and identify strategies for promoting resilience among already at-risk children.

### Potential moderating factors

In adolescence and adulthood, BPD is characterized by both high impulsivity and high lability in negative affect, contributing to the high rates of self-injurious and risk-taking behaviors in this population [1]. Several studies have identified caregiver- and teacher-reported impulsivity and emotional lability in early and late childhood to be prospectively associated with later BPD symptoms [18, 19, 23–25], and other work suggests that impulsivity and emotional lability may interact with one another or with other risk factors to exacerbate BPD risk. Stepp, Whalen, and Pedersen [26], for example, proposed that engaging in impulsive behaviors when experiencing intense emotions may be a critical developmental precursor of BPD. It follows, then, that children who demonstrate lower impulsivity in response to heightened emotional intensity, or who are less likely to experience intense negative emotions in the first place, may be at less risk for developing BPD. Belsky [27] and Belsky and Pluess [28] also offered the differential susceptibility hypothesis, asserting that children with lower negative emotionality or lower impulsivity are less susceptible to the effects of childhood adversity on psychopathology, demonstrating fewer psychopathology symptoms in childhood. Children with high ACEs who demonstrate low impulsivity or low emotional lability, then, might avoid added BPD risk conferred by psychopathology symptoms.

Initiative-taking, which we define as engaging in active coping strategies and seeking out resources in order to meet goals, has received less attention in the BPD literature. Various studies have investigated the role of childhood social support and peer relationships in the development of BPD, with mixed results [10, 19, 21], but Paris et al. [21] suggest that the apparent protective effects of social support may actually reflect the benefits of initiative-taking more broadly. Traditionally, research on initiative-taking has focused on intrapersonal processes such as problem solving and cognitive reappraisal, but recent research suggests that interpersonal processes—including seeking out social support to enhance one’s own happiness, reduce distress, and model others’ coping strategies—should also be considered part of the active coping strategy repertoire [29–31]. Use of intrapersonal active coping strategies has been shown to be associated with positive academic, social, and personal-emotional adjustment to college [32] and fewer depressive symptoms, specifically in the face of high stress [33]. Further, use of interpersonal active coping strategies has been shown to be associated with developing more supportive relationships [34] and greater resilience in the face of adversity [35]. Prior work suggests that adolescents and adults with BPD tend to have deficits in intrapersonal coping [36], such as decreased use of cognitive reappraisal [37] and acceptance [38, 39] coping strategies; higher intrapersonal initiative-taking in childhood, then, might help children expand their active coping repertoire early on and mitigate the impact of emotion dysregulation on the BPD development trajectory. As summarized by Sharp and Vanwoerden [40], interpersonal deficits (such as mentalizing impairments, increased rejection sensitivity, and increased relational conflict) are also a key feature of BPD; therefore it is worth exploring whether the ability to engage in interpersonal coping in childhood might help foster healthier interpersonal patterns and help mitigate the development of these interpersonal deficits. Few studies have examined if greater use of interpersonal and intrapersonal coping strategies in early childhood can support better coping in adolescence and/or reduce future BPD symptoms, and as such, the current study seeks to address this gap in the literature.

### Observational measures

In addition to the gap in the literature concerning moderating factors in BPD development, there are also few studies that have employed observational methods to measure aspects of temperament and behavior that might serve as risk and protective factors for BPD. Both parent and observer reports of child temperament and behavior have been shown to demonstrate temporal stability [41] and predictive validity [42], and compared to parent- or self-report, observational measures of temperament are more objective and standardized across participants. For example, behavioral coding of in-laboratory tasks can be standardized by using specific behavior rating manuals and by monitoring interrater reliability, whereas parent- and self-report can be biased by subjective perceptions, interpretations, and ideas about norms of behavior [42, 43]. Keeping in mind that observational measures can also present unique challenges, such as their cross-sectional nature and questions about ecological validity, the current study adds to the literature by including both observed and parent-reported measures for each hypothesized moderator.

### The current study

The goal of the current study was to identify individual-level preschool-age factors that moderate the relationship between childhood adversity and the development of adolescent BPD or moderate the relationship between childhood psychopathology and the development of adolescent BPD. Noting the reliance on retrospective, self-reported data in prior BPD protective factors research, we assessed moderating factors prospectively utilizing both observer and parent reports. We also sought to contribute to the literature on early childhood factors in BPD development by assessing risk and moderating factors in the preschool period (ages 3–5).

We hypothesized that parent-reported and observed preschool impulsivity, emotional lability, and initiative-taking would moderate the relationship between preschool risk factors and BPD symptoms in adolescence. Specifically, we predicted that preschool children who had high ACEs, but also demonstrated lower impulsivity, lower emotional lability, or higher initiative-taking, would have fewer BPD symptoms in adolescence than peers exposed to high ACEs who demonstrated higher impulsivity, higher emotional lability, or lower initiative-taking in preschool. We also expected each of these factors to similarly moderate the relationship between preschool internalizing and externalizing symptoms and BPD symptoms in adolescence.

## Methods

Participants were 151 children who were enrolled in the longitudinal Preschool Depression Study at the Washington University School of Medicine in St. Louis Early Emotional Development Program, which prospectively investigated associations between childhood factors and later outcomes. Participants were initially recruited during the preschool period (ages 3–6) from daycare and primary care centers in the St. Louis region, and the final sample of 306 children was selected after oversampling for higher depressive and disruptive the symptoms based on scores from the Preschool Feelings Checklist [44]. Participants completed up to two preschool assessments between the ages of 3 and 6 years old and up to two adolescent assessments between the ages of 14 and 21 (see Table 1 for descriptive statistics), in addition to other intervening assessments not reported on in the current study. The 151 children included in the present study are those of the original sample who completed assessments for all preschool risk factors, at least one moderating factor assessment, and an assessment of BPD symptoms at least once during adolescence. The participants who completed an adolescent assessment were not statistically different in terms of preschool age (*t* = -1.04, *p* = .30), ACEs (*t* = -1.78, *p* = .08), average internalizing symptoms (*t* = -0.20, *p* = 0.84), average externalizing symptoms (*t* = .79, *p* = .43), parent-reported (*t* = -.59, *p* = .56) or observed impulsivity (*t* = -0.26, *p* = .80), parent-reported (*t* = -0.18, *p* = .86) or observed emotional lability (*t* = -0.22, *p* = .83), or parent-reported (*t* = 0.98, *p* = .33) or observed initiative-taking (*t* = 0.00, *p* = .997) from participants in the original sample who did not complete this assessment.

**Table 1:** Descriptive statistics of predictor, moderator, and outcome variables and ages

Variable	*n*	Range	*M*	*SD*	Skewness	Kurtosis	Tolerance	VIF
Time 1 ACEs	151	0.0–10	2.96	2.03	.87	.59	.746	1.340
Time 1 internalizing	151	0.0–15.0	2.21	2.90	1.94	3.92	.731	1.367
Time 1 externalizing	151	0.0–31.0	7.06	6.75	1.27	1.14	.438	2.283
Obs. impulsivity	151	1.45–3.63	2.31	0.37	0.42	0.34	.695	1.439
Par. impulsivity	149	0.0–2.0	0.83	0.41	0.49	0.25	.453	2.206
Obs. emotional lability	143	0.0–3.79	0.78	0.75	1.51	2.74	.713	1.402
Par. emotional lability	149	0.0–9.50	3.13	2.02	0.54	-0.11	.462	2.167
Obs. initiative-taking	151	1.33–3.24	2.29	0.36	-0.16	-0.08	.676	1.480
Par. initiative-taking	148	-1.42–0.0	-0.59	0.32	-0.26	-0.25	.820	1.219
Maximum BPFS-C score	151	27.0–104.0	60.77	14.39	0.29	-0.26		
Preschool time 1 age	151	3–6	4.50	0.79				
Preschool time 2 age	146	4–6	5.52	0.78				
Adolescent time 1 age	136	14–19	16.44	0.98				
Adolescent time 2 age	137	16–21	18.69	1.06				

*Note*: Range for variables represents the range of participants’ actual scores. Ages are in years. *Obs.* Observed, *Par.* Parent-reported, *VIF* Variance inflation factor

### Assessments of early childhood risk factors

Our choice of early childhood risk factors was based on prior work by Geselowitz et al. [10], who reported on a subset of the Preschool Depression Study, but our sample was further restricted to participants who had completed an assessment at which at least one preschool moderating factor was measured.

#### Adverse childhood experiences

Consistent with past work in this sample, we used an ACEs measure described by Luby et al. [45] and Barch et al. [46] that was developed for our wider longitudinal study based on the ACEs construct proposed by Felitti et al. [47]. We constructed a sum variable for parent-reported ACEs at each assessment from preschool through adolescence. The number of non-redundant traumatic events (including abuse, serious accidents and disasters, parental arrest or hospitalization, death of a loved one, and similar threatening events) experienced or witnessed by the participant were summed and added to: parental suicide attempt (1 if present), parental substance abuse (1 if present), parental psychopathology (1 if present), and living below the poverty line (1 if present). We included all available ACEs sums from preschool through adolescence, which included up to 10 assessments, as variables in our analyses (*M* = 8.66, *SD* = 1.10, Range = 5–10).

#### Child psychopathology

Our internalizing psychopathology measure was calculated by summing symptom scores assessing generalized anxiety disorder, separation anxiety, and post-traumatic stress disorder from the parent-reported Preschool Age Psychiatric Assessment (PAPA) [48] at each preschool assessment. Our externalizing psychopathology measure was calculated by summing symptom scores assessing attention-deficit/ hyperactivity disorder (ADHD), oppositional defiant disorder, and conduct disorder from the PAPA at each preschool assessment. The PAPA is an empirically validated, interviewer-administered clinical diagnostic instrument designed for use in children between the ages of 2 and 6 years [49]. To maintain interviewer reliability and calibration, a master interviewer reviewed approximately 20% of the audiotaped PAPA interviews and held weekly meetings with other interviewers. When discrepancies were found during review, interviews were re-coded in consultation with a senior child psychiatrist (as recommended by the authors of the PAPA interview). We included two internalizing sums and two externalizing sums as variables in our analyses, one for each preschool assessment.

### Assessments of early childhood moderating factors

Observer ratings of early childhood moderating factors were obtained through coding of Preschool Laboratory Temperament Assessment Battery (Lab-TAB) tasks [50]. Lab-TAB tasks are short, standardized, experimenter-guided episodes designed to elicit emotions and behaviors in children and allow for assessments of various dimensions of early childhood temperament [51], with moderate test–retest reliability and construct validity [41, 42, 52].

Participants in the current study completed at least one of four Lab-TAB tasks during preschool assessments: two excitement-inducing tasks and two frustration-inducing tasks. In brief, the tasks proceeded as follows: (1) *Make the Car Go*: the child and experimenter raced wind-up cars on a model racetrack; (2) *Popping Bubbles*: the child blew bubbles with a bubble gun and popped the bubbles; (3) *I’m Not Sharing*: the experimenter did not share toys with the child and took more and more of the child’s toys; (4) *Empty Box*: the child was left alone to open a wrapped gift box only to discover there was no gift inside. At the end of each frustration task, the experimenter rectified the situation by apologizing and giving the child toys or a new present.

Teams of five to six research assistants blind to the child’s diagnostic characteristics coded videotapes of each task, rating participants’ facial, verbal, and bodily expressions of affect and rating behavioral dimensions including impulsivity, initiative, sociability, and activity level. Ratings were given based on a coding guide developed and adapted from the original preschool Lab-TAB manual [50] by researchers at the Washington University School of Medicine in St. Louis Early Emotional Development Program, which specified criteria for each rating on a 4-point Likert scale. Criteria accounted for both intensity and frequency of emotional expressions and behaviors. Coders were required to reach at least 80% agreement with an expert coder during training before coding independently and interrater reliability was assessed weekly during coding to ensure reliability of at least 80%.

Parent ratings of early childhood moderating factors were obtained from the MacArthur Health and Behavior Questionnaire—Parent Version (HBQ) [53] during the first preschool assessment. The HBQ is a parent-reported assessment of physical and mental health symptoms and behaviors, as well as social and school functioning, in children 4 to 8 years old [54] that has demonstrated high test–retest reliability and construct validity for assessing early childhood psychopathology [55–57].

#### Impulsivity

Observed impulsivity was calculated as an average of observer-rated impulsivity and activity level across the four Lab-TAB tasks and both preschool assessments. Higher impulsivity was defined as acting without thinking, including interrupting, invading others’ personal space, and grabbing objects. Activity level was rated on a scale between sitting completely still and constantly moving. These definitions are comparable to the DSM-5 hyperactivity/impulsivity criteria for ADHD diagnosis [22] and to measures used in prior work examining early childhood impulsivity [19, 58]. We included activity level in our impulsivity measure given prior research suggesting that impulsivity and hyperactivity are interrelated in the preschool period and are not easily separable without more extensive measures of each construct [59].

Parent-reported impulsivity was calculated as an average of HBQ impulsivity subscale scores across preschool assessments. Items on this subscale assess both inhibitory control and hyperactivity. The HBQ’s impulsivity and inattention subscales have demonstrated high validity in terms of identifying children with clinical symptoms of ADHD [57].

#### Emotional lability

Observed emotional lability was calculated as a function of observer-rated negative affect (anger and sadness) from the two frustration-eliciting Lab-TAB tasks. These tasks were coded in segments, such that one code for each dimension of affect was given in each of the following periods: before the frustrating event occurred, after the frustrating event occurred, and after the situation was rectified. Anger/frustration criteria included tense facial features, forceful body movements, yelling, and angry verbal content. Sadness criteria included downturned mouth, slumped shoulders, crying, and sad verbal content.

In order to quantify observed emotional lability, we sought to assess the amount of change in negative affect over the course of the frustration tasks. We decided to use the mean of squared successive differences (MSSD) approach, which in past work has been used to quantify emotional lability using self-reported negative affect [60, 61]. First, we calculated composite ratings of angry and sad affect within each task segment by taking an average across facial, verbal, and bodily affect ratings. We then calculated squared successive differences (SSDs) in these composite ratings between consecutive task sections (differences in composite anger between segment 1 and segment 2, then between segment 2 and segment 3), keeping the two tasks separate and anger and sadness separate. We then calculated an average of all SSDs across all tasks and across anger and sadness; this comprised our MSSD and our final observed emotional lability variable.

Parent-reported emotional lability was assessed with items chosen from the depression and oppositional-defiant subscales of the HBQ, leading to sum scores for each preschool assessment of which we took the average to create a final variable. The items chosen were: ‘unhappy, sad, or depressed,’ ‘cries a lot,’ ‘has temper tantrums or hot temper,’ ‘is easily annoyed by others,’ and ‘angry and resentful.’ These items were chosen to reflect both the negative emotionality and affective lability components of the emotional lability construct.

#### Initiative-taking

Observed initiative-taking was calculated as an average of observer-rated initiative and sociability across the four Lab-TAB tasks and both preschool assessments. Higher initiative during the task was defined as making suggestions for play, asking for a turn, and trying to rectify an unfair situation. This measure represents intrapersonal active coping and is consistent with prior work that operationalized preschool initiative as self-starting, information seeking, and persisting during problem solving [62]. Higher sociability was defined as interacting with the experimenter, including turn-taking in speech and body language and making eye contact, which emphasized the child’s role in taking action to engage with the experimenter and allowed us to assess interpersonal active coping.

Parent-reported initiative-taking was calculated as an average of HBQ social withdrawal subscale scores across preschool assessments. This subscale assesses the extent to which the child avoids, withdraws from, or is shy around peers and adults. We reverse coded scores so that children with higher social withdrawal scores were coded as lower on initiative-taking. Assessments of parent-reported initiative-taking were limited in the Preschool Depression Study, and while we recognize that this subscale is not a comprehensive measure of initiative-taking, this measure could clarify whether interpersonal coping alone plays a protective role against BPD development, or if both intrapersonal and interpersonal coping strategies are necessary.

### Adolescent outcome measures

#### BPD symptoms

Adolescents (ages 14–21) self-reported BPD symptoms using the Borderline Personality Features Scale for Children (BPFS-C) [63], which has demonstrated high criterion validity [64]. Scores on the BPFS-C range from 24 to 120, with higher scores indicating more BPD symptoms. We used a continuous measure of BPFS-C scores in our analyses, and when participants had completed the BPFS-C at two adolescent assessments, we used the maximum of their two scores as the BPD symptom measure. To aid in interpretation of results, we considered the empirically determined, clinically relevant BPFS-C cut-off score of 65 to understand which participants might qualify for a presumptive BPD diagnosis [64], but this was not used in our analyses.

### Plan of analyses

#### Examining risk factors

First, in order to replicate past associations between preschool risk factors and adolescent BPD symptoms found by Geselowitz et al. [10] using a slightly different subset of the wider Preschool Depression Study, we ran similar analyses to confirm these predictions were also significant in our sample. Specifically, we used a multilevel linear modeling framework with measurements of the risk factors across time nested within individuals to examine the association between initial scores of three preschool risk factors (i.e., ACEs, internalizing symptoms, and externalizing symptoms) with future adolescent BPD symptom scores. An example equation can be seen below:

- Level 1:
  $${RF}_{ij}= {b}_{0j}+ {b}_{1j}{time0}_{ij}+ {e}_{ij}$$
- Level 2:
  $${b}_{0j}= {\gamma }_{00}+ {\gamma }_{10}{age.c}_{j}+ {\gamma }_{20}{sex}_{j}+ {\gamma }_{30}{BPFSC.z}_{j}+ {U}_{0j}$$
  $${b}_{1j}= {\gamma }_{10}$$

In order to leverage the nested measurements of each risk factor (${RF}_{ij}$) across time and quantify the association between the initial measurement of each risk factor and future BPD symptom scores, each risk factor was treated as the outcome variable and future BPD symptom scores were treated as a Level 2 predictor variable. Time was centered around the initial assessment wave and scaled according to the number of the measurement occasion, age was centered around the average age at the initial timepoint, sex was a dichotomous variable with effect coding (-1 = male, 1 = female), and BPD symptom scores were standardized. Thus, ${b}_{0j}$ represents the average initial score for each risk factor; ${b}_{1j}$ represents the amount of change in each risk factor across each assessment wave; ${\gamma }_{10}$ represents the effect of deviating from the average age at the initial timepoint for each risk factor score; ${\gamma }_{20}$ represents the effect of sex (i.e., subtract from the intercept for males, add to the intercept for females); and ${\gamma }_{30}$ represents the magnitude of the association between the initial scores for each risk factor and a one-unit (i.e., one standard deviation) change in future BPFSC scores. The ${\gamma }_{30}$ parameter from each model was of main interest. A total of three models were run, one for each risk factor.

#### Correlations

We examined the zero-order correlations between the observer- and parent-reported measures of each proposed moderating variable. This was done to ensure distinct variance would be captured with the multi-method variables when they were incorporated into the multilevel models. We further examined the zero-order correlations between risk factor and moderator variables to assess construct relatedness.

#### Examining moderating factors

We then included the observer- and parent-reported moderating variables in the analyses. The observer- and parent-report variables for each construct were included in the same model to examine the unique associations of each method report with our variables of interest. An example equation that builds off of the prior equation can be seen below:

- Level 1:
  $${RF}_{ij}= {b}_{0j}+ {b}_{1j}{time0}_{ij}+ {e}_{ij}$$
- Level 2:
  $${b}_{0j}= {\gamma }_{00}+ {\gamma }_{10}{age.c}_{j}+ {\gamma }_{20}{sex}_{j}+ {\gamma }_{30}{BPFSC.z}_{j}+ {\gamma }_{40}{Mod.P.z}_{j}+ {\gamma }_{50}{Mod.O.z}_{j}+ {\gamma }_{60}{Mod.P.z*BPFSC.z}_{j}+ {\gamma }_{70}{Mod.O.z*BPFSC.z}_{j}+ {U}_{0j}$$
  $${b}_{1j}= {\gamma }_{10}$$

The variables for time, age, sex, and BPD symptom scores were equivalent to those in the previous set of models. The parent- (${Mod.P.z}_{j}$) and observer-reported (${Mod.O.z}_{j}$) moderating variables were standardized and treated as Level 2 predictor variables. Thus, ${b}_{0j}$ represents the average initial score for each risk factor; ${b}_{1j}$ represents the amount of change in each risk factor across each assessment wave; ${\gamma }_{10}$ represents the effect of deviating from the average age at the initial timepoint for each risk factor score; ${\gamma }_{20}$ represents the effect of sex; ${\gamma }_{30}$ represents the magnitude of the association between the initial scores for each risk factor and a one-unit (i.e., one standard deviation) change in future BPFSC scores, for an individual with average scores on each moderating variable; ${\gamma }_{40}$ represents the magnitude of the association between the initial scores for each risk factor and a one-unit change in the parent-reported moderating variable, for an individual with average BPD symptom and observer-reported moderating variable scores; ${\gamma }_{50}$ represents the magnitude of the association between the initial scores for each risk factor and a one-unit change in the observer-reported moderating variable, for an individual with average BPD symptom and parent-reported moderating variable scores; ${\gamma }_{60}$ represents the magnitude of the association between one-unit changes on both the parent-reported moderating variable and future BPD symptom scores with the initial risk factor; and ${\gamma }_{70}$ represents the magnitude of the association between one-unit changes on both the observer-reported moderating variable and future BPD symptom scores with the initial risk factor. The ${\gamma }_{60}$ and ${\gamma }_{70}$ parameters from each model were of main interest. A total of nine models were run, three for each risk factor with each set of parent- and observer-reported moderating variables (i.e., impulsivity, initiative-taking, and lability).

In order to compensate for the number of models (12 total), the False Discovery rate (FDR) correction was applied to all analyses except correlations (corrected *p*-values are indicated with *p*_*corrected*_). All analyses were conducted using R statistical software, version 4.2.0 [65] using the lme4 package [66]. Code for all analyses is included in the accompanying supplementary material (Additional File 1).

## Results

### Demographic characteristics

Our sample included 151 participants, including 76 female participants and 75 male participants. Participants’ maximum BPFS-C scores ranged from 27 to 104; possible scores on the BPFS-C range from 24 to 120. Using the BPFS-C cut-off score of 65, fifty-eight of the participants (38.4%) qualified for a presumptive BPD diagnosis during at least one adolescent assessment. The majority of participants completed at least one assessment for all moderator variables. See Table 1 for descriptive statistics of study variables and ages at assessments.

### Baseline predictions

Each of our three risk factors assessed at the initial timepoint (an average of over 13 years later), were significantly associated with future BPD symptom scores, controlling for age, sex, and changes in the risk factors across time (Table 2). More specifically, a one standard deviation increase in BPD symptom scores was associated with higher initial ACEs (*b* = 0.52, 95% CI [0.30, 0.73], *p*_*corrected*_ < .001), internalizing symptoms (*b* = 0.76, 95% CI [0.39, 1.12], *p*_*corrected*_ < .001), and externalizing symptoms (*b* = 1.65, 95% CI [0.72, 2.57], *p*_*corrected*_ = .002).

**Table 2:** Baseline predictions

Model	Effect	Variable	Est	95% CI	*t*	*p*	*FDR-p*
ACEs							
	Random						
		Intercept	1.25	[1.07, 1.41]			
	Fixed						
		Intercept	**1.68**	**[1.44, 1.93]**	**13.31**	**.000**	**.000**
		Time	**0.21**	**[0.18, 0.23]**	**15.03**	**.000**	**.000**
		Age	0.08	[-0.20, 0.35]	0.53	.598	.726
		Sex	0.01	[-0.21, 0.23]	0.08	.935	.965
		BPFSC (z)	**0.52**	**[0.30, 0.73]**	**4.59**	**.000**	**.000**
	Error						
		Residual	1.60	[1.54, 1.67]			
Internalizing							
	Random						
		Intercept	1.76	[1.36, 2.09]			
	Fixed						
		Intercept	**2.21**	**[1.78, 2.63]**	**10.09**	**.000**	**.000**
		Time	-0.23	[-0.70, 0.23]	-0.99	.325	.511
		Age	0.34	[-0.12, 0.81]	1.44	.151	.279
		Sex	-0.13	[-0.50, 0.24]	-0.68	.495	.634
		BPFSC (z)	**0.76**	**[0.39, 1.12]**	**4.04**	**.000**	**.000**
	Error						
		Residual	2.03	[1.81, 2.28]			
Externalizing							
	Random						
		Intercept	5.18	[4.42, 5.89]			
	Fixed						
		Intercept	**7.07**	**[6.08, 8.06]**	**13.88**	**.000**	**.000**
		Time	**-1.35**	**[-2.15, -0.54]**	**-3.28**	**.001**	**.004**
		Age	1.18	[0.00, 2.35]	1.95	.053	.113
		Sex	-1.11	[-2.04, -0.18]	-2.32	.022	.052
		BPFSC (z)	**1.65**	**[0.72, 2.57]**	**3.47**	**.001**	**.002**
	Error						
		Residual	3.50	[3.12, 3.93]			

*Note*. *Est* model-provided parameter estimate. *CI* Confidence interval. *t* t-test statistic, *p p-*value, *FDR-p* FDR-corrected *p*-value. Bolded values indicate those that are significant at FDR-corrected *p* < .05. The “(z)” after a variable indicates it is standardized

### Associations between parent- and observer-reported moderators

Next, we examined the magnitude of the association between each of the parent- and observer-reported moderating variables. The correlations varied from insignificant to small. Observer- and parent-reported impulsivity were positively correlated (*r*(149) = .24, *p* = .004) as were observer- and parent-reported initiative-taking (*r*(148) = .21, *p* = .01). Observer-reported emotional lability was not significantly correlated with parent-reported emotional lability (*r*(141) = .03, *p* = .72). Correlations between study variables are shown in Table 3.

**Table 3:** Correlations between study variables

	1	2	3	4	5	6	7	8
1. T1 ACEs								
2. T1 Internalizing	.324^**^							
3. T1 Externalizing	.427^**^	.453^**^						
4. Obs. impulsivity	.130	.029	.187^*^					
5. Par. impulsivity	.398^**^	.321^**^	.627^**^	.236^**^				
6. Obs. lability	-.010	-.110	.047	.393^**^	.100			
7. Par. lability	.284^**^	.349^**^	.597^**^	.105	.631^**^	.030		
8. Obs. initiative	.063	-.056	.020	.420^**^	.122	.461^**^	-.011	
9. Par. initiative	.058	-.137	.028	.104	.000	.212^*^	-.230^**^	.205^*^

*Note*: Values in table are *r*-values. *N*-values for correlations ranged from 137 to 151. *p < .05. **p < .01. T1 = preschool time 1

*Obs.* Observed, *Par.* Parent-reported

### Predictions with moderators

Lastly, we examined if any of our parent- and observer-reported measures moderated the association between the initial risk factors and future BPD symptom scores. For the models including initial ACEs, no significant interactions emerged (Table 4). ACEs were still significantly associated with future BPD symptom scores across all three models while controlling for moderating variables. Additionally, parent-reported impulsivity (*b* = 0.70, 95% CI [0.48, 0.91], *p*_*corrected*_ < .001) and parent-reported lability (*b* = 0.58, 95% CI [0.36, 0.80], *p*_*corrected*_ < .001) were significantly associated with initial ACEs scores.

**Table 4:** ACEs and Continuous BPD Symptoms

Model	Effect	Variable	Est	95% CI	*t*	*p*	*FDR-p*
Impulsivity							
	Random						
		Intercept	1.06	[0.88, 1.18]			
	Fixed						
		Intercept	**1.65**	**[1.43, 1.88]**	**14.07**	**.000**	**.000**
		Time	**0.21**	**[0.18, 0.23]**	**14.87**	**.000**	**.000**
		Age	-0.09	[-0.34, 0.16]	-0.71	.480	.623
		Sex	0.08	[-0.12, 0.28]	0.77	.445	.607
		BPFSC (z)	**0.36**	**[0.16, 0.56]**	**3.52**	**.001**	**.002**
		Parent Impulsivity (z)	**0.70**	**[0.48, 0.91]**	**6.26**	**.000**	**.000**
		Obs Impulsivity (z)	-0.02	[-0.22, 0.18]	-0.21	.834	.931
		BPFSC (z) x Parent Impulsivity (z)	0.00	[-0.21, 0.21]	0.00	.998	.998
		BPFSC (z) x Obs Impulsivity (z)	0.16	[-0.05, 0.37]	1.45	.150	.279
	Error						
		Residual	1.61	[1.55, 1.68]			
Initiative							
	Random						
		Intercept	1.23	[1.04, 1.37]			
	Fixed						
		Intercept	**1.65**	**[1.40, 1.89]**	**12.87**	**.000**	**.000**
		Time	**0.20**	**[0.18, 0.23]**	**14.68**	**.000**	**.000**
		Age	0.13	[-0.15, 0.40]	0.88	.379	.560
		Sex	-0.01	[-0.23, 0.20]	-0.12	.907	.965
		BPFSC (z)	**0.53**	**[0.31, 0.75]**	**4.72**	**.000**	**.000**
		Parent Initiative (z)	0.03	[-0.20, 0.26]	0.23	.821	.931
		Obs Initiative (z)	-0.01	[-0.23, 0.21]	-0.09	.926	.965
		BPFSC (z) x Parent Initiative (z)	-0.07	[-0.31, 0.17]	-0.58	.562	.692
		BPFSC (z) x Obs Initiative (z)	0.18	[-0.04, 0.41]	1.55	.124	.243
	Error						
		Residual	1.61	[1.54, 1.68]			
Lability							
	Random						
		Intercept	1.11	[0.92, 1.25]			
	Fixed						
		Intercept	**1.61**	**[1.37, 1.85]**	**12.77**	**.000**	**.000**
		Time	**0.21**	**[0.18, 0.24]**	**14.79**	**.000**	**.000**
		Age	-0.02	[-0.28, 0.23]	-0.18	.854	.942
		Sex	-0.01	[-0.21, 0.20]	-0.05	.960	.970
		BPFSC (z)	**0.35**	**[0.14, 0.56]**	**3.18**	**.002**	**.005**
		Parent Lability (z)	**0.58**	**[0.36, 0.80]**	**5.02**	**.000**	**.000**
		Obs Lability (z)	0.01	[-0.19, 0.22]	0.12	.906	.965
		BPFSC (z) x Parent Lability (z)	0.08	[-0.12, 0.28]	0.76	.451	.607
		BPFSC (z) x Obs Lability (z)	0.01	[-0.20, 0.22]	0.09	.929	.965
	Error						
		Residual	1.62	[1.55, 1.69]			

*Note*. *Est* Model-provided parameter estimate, *CI* Confidence interval. *t* t-test statistic, *p p-*value, *FDR-p* FDR-corrected *p*-value. *Obs* observed. Bolded values indicate those that are significant at FDR-corrected *p* < .05. The “(z)” after a variable indicates it is standardized

**Fig. 1:**
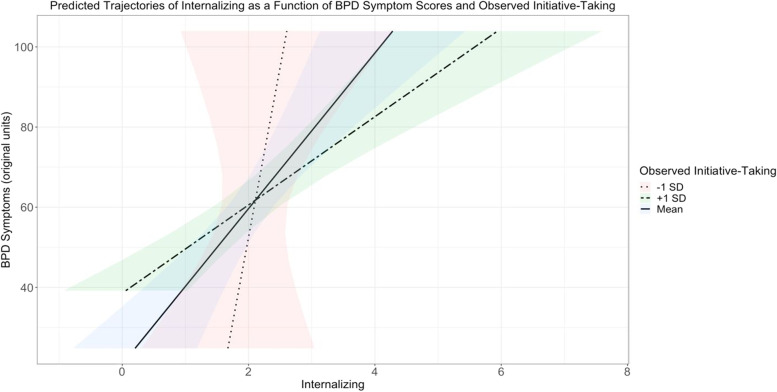
Predicted trajectories of internalizing as a function of BPD symptom scores and observed initiative-taking

For the models including initial internalizing symptom scores, internalizing was still associated with future BPD symptom scores while controlling for each of the moderating variables (Table 5). Additionally, in the models with impulsivity and lability, main effects emerged such that initial internalizing symptom scores were positively associated with parent-reported impulsivity (*b* = 0.61, 95% CI [0.21, 1.01], *p*_*corrected*_ = .011) and parent-reported lability (*b* = 0.81, 95% CI [0.43, 1.20], *p*_*corrected*_ < .001). Then, in the model with initiative-taking, an interaction emerged (Fig. 1). This interaction indicated that the effect of a child’s initial internalizing symptom score on their future BPD symptom score varied as a function of their observed initiative-taking score (*b* = 0.57, 95% CI [0.20, 0.95], *p*_*corrected*_ = .011). For children scoring high on internalizing, scoring one standard deviation higher than average on observer-reported initiative-taking appeared to be protective, as their BPD symptom scores were lower than those scoring average or lower than average on observer-reported initiative-taking. In comparison, for children scoring low on internalizing, having higher than average observer-reported initiative-taking scores was associated with higher BPD symptom scores compared to children scoring average or below average on observer-reported initiative-taking.

**Table 5:** Internalizing and continuous BPD symptoms

Model	Effect	Variable	Est	95% CI	*t*	*p*	*FDR-p*
Impulsivity							
	Random						
		Intercept	1.69	[1.23, 1.98]			
	Fixed						
		Intercept	**2.16**	**[1.73, 2.58]**	**9.76**	**.000**	**.000**
		Time	-0.25	[-0.72, 0.22]	-1.04	.302	.483
		Age	0.19	[-0.28, 0.66]	0.78	.434	.607
		Sex	-0.05	[-0.43, 0.32]	-0.26	.792	.916
		BPFSC (z)	**0.60**	**[0.24, 0.97]**	**3.15**	**.002**	**.006**
		Parent Impulsivity (z)	**0.61**	**[0.21, 1.01]**	**2.93**	**.004**	**.011**
		Obs Impulsivity (z)	0.01	[-0.36, 0.38]	0.06	.954	.970
		BPFSC (z) x Parent Impulsivity (z)	0.12	[-0.27, 0.50]	0.59	.559	.692
		BPFSC (z) x Obs Impulsivity (z)	0.25	[-0.14, 0.64]	1.22	.223	.382
	Error						
		Residual	2.04	[1.82, 2.29]			
Initiative							
	Random						
		Intercept	1.69	[1.23, 1.99]			
	Fixed						
		Intercept	**2.20**	**[1.78, 2.62]**	**10.00**	**.000**	**.000**
		Time	-0.26	[-0.74, 0.21]	-1.08	.280	.464
		Age	0.47	[0.01, 0.93]	1.95	.053	.113
		Sex	-0.13	[-0.49, 0.23]	-0.72	.473	.622
		BPFSC (z)	**0.72**	**[0.37, 1.08]**	**3.88**	**.000**	**.001**
		Parent Initiative (z)	-0.37	[-0.76, 0.01]	-1.87	.064	.134
		Obs Initiative (z)	-0.04	[-0.40, 0.32]	-0.21	.834	.931
		BPFSC (z) x Parent Initiative (z)	0.02	[-0.38, 0.41]	0.09	.932	.965
		BPFSC (z) x Obs Initiative (z)	**0.57**	**[0.20, 0.95]**	**2.92**	**.004**	**.011**
	Error						
		Residual	2.05	[1.83, 2.30]			
Lability							
	Random						
		Intercept	1.66	[1.21, 1.95]			
	Fixed						
		Intercept	**2.13**	**[1.71, 2.56]**	**9.61**	**.000**	**.000**
		Time	-0.25	[-0.72, 0.21]	-1.07	.285	.465
		Age	0.21	[-0.23, 0.66]	0.91	.362	.552
		Sex	-0.15	[-0.51, 0.21]	-0.82	.412	.599
		BPFSC (z)	**0.51**	**[0.15, 0.88]**	**2.68**	**.008**	**.021**
		Parent Lability (z)	**0.81**	**[0.43, 1.20]**	**4.07**	**.000**	**.000**
		Obs Lability (z)	-0.25	[-0.61, 0.10]	-1.37	.173	.308
		BPFSC (z) x Parent Lability (z)	0.27	[-0.08, 0.61]	1.46	.147	.279
		BPFSC (z) x Obs Lability (z)	0.15	[-0.23, 0.52]	0.75	.455	.607
	Error						
		Residual	1.95	[1.73, 2.20]			

*Note*. *Est* Model-provided parameter estimate, *CI* Confidence interval, *t* t-test statistic, *p p-*value, *FDR-p* FDR-corrected *p*-value, *Obs* observed. Bolded values indicate those that are significant at FDR-corrected *p* < .05. The “(z)” after a variable indicates it is standardized

**Fig. 2:**
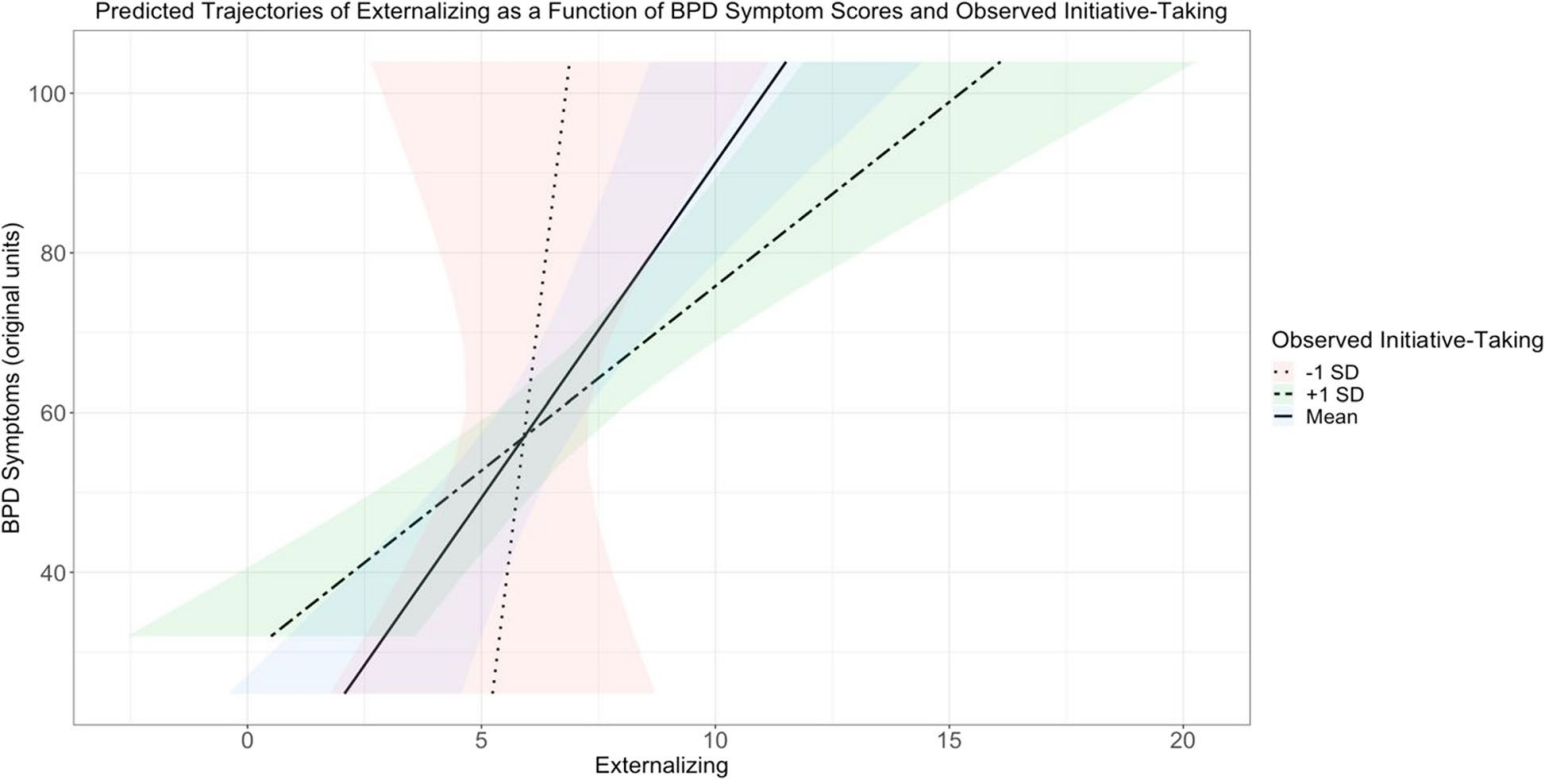
Predicted trajectories of externalizing as a function of BPD symptom scores and observed Initiative-taking

Lastly, for the models including initial externalizing symptom scores, a number of significant associations emerged (Table 6). In the models with impulsivity and lability, main effects emerged such that initial externalizing symptom scores were positively associated with parent-reported impulsivity (*b* = 4.13, 95% CI [3.37, 4.89], *p*_*corrected*_ < .001) and parent-reported lability (*b* = 3.74, 95% CI [2.91, 4.57], *p*_*corrected*_ < .001). However, in contrast to the baseline models, childhood externalizing was no longer associated with future BPD symptom scores when controlling for impulsivity measures (*b* = 0.64, 95% CI [-0.06, 1.33], *p*_*corrected*_ = .166) or emotional lability measures (*b* = 0.57, 95% CI [-0.22, 1.37], *p*_*corrected*_ = .303). In the model with initiative-taking, childhood externalizing was once again significantly associated with future BPD symptoms (*b* = 1.66, 95% CI [0.75, 2.56], *p*_*corrected*_ = .002), and a similar interaction effect emerged to that found for internalizing symptoms (Fig. 2). This interaction indicated that the effect of a child’s initial externalizing symptom score on their future BPD symptom score varied as a function of their observed initiative-taking score (*b* = 1.42, 95% CI [0.46, 2.37], *p*_*corrected*_ = .013). For children scoring high on externalizing, scoring one standard deviation higher than average on observer-reported initiative-taking again appeared to be protective. In comparison, for children scoring low on externalizing, having higher than average observer-reported initiative-taking scores was associated with higher BPD symptom scores compared to children scoring average or below average on this variable.

**Table 6:** Externalizing and continuous BPD symptoms

Model	Effect	Variable	Est	95% CI	*t*	*p*	*FDR-p*
Impulsivity							
	Random						
		Intercept	3.41	[2.65, 3.93]			
	Fixed						
		Intercept	**6.96**	**[6.17, 7.74]**	**17.00**	**.000**	**.000**
		Time	**-1.38**	**[-2.19, -0.57]**	**-3.33**	**.001**	**.004**
		Age	0.20	[-0.68, 1.08]	0.43	.666	.789
		Sex	-0.34	[-1.05, 0.38]	-0.90	.369	.553
		BPFSC (z)	0.64	[-0.06, 1.33]	1.75	.083	.166
		Parent Impulsivity (z)	**4.13**	**[3.37, 4.89]**	**10.43**	**.000**	**.000**
		Obs Impulsivity (z)	0.35	[-0.36, 1.06]	0.94	.346	.536
		BPFSC (z) x Parent Impulsivity (z)	0.76	[0.03, 1.49]	1.99	.049	.109
		BPFSC (z) x Obs Impulsivity (z)	0.45	[-0.29, 1.19]	1.16	.248	.418
	Error						
		Residual	3.52	[3.13, 3.95]			
Initiative							
	Random						
		Intercept	5.08	[4.23, 5.69]			
	Fixed						
		Intercept	**7.00**	**[6.02, 7.99]**	**13.66**	**.000**	**.000**
		Time	**-1.42**	**[-2.24, -0.60]**	**-3.40**	**.001**	**.003**
		Age	1.34	[0.18, 2.51]	2.21	.029	.066
		Sex	**-1.14**	**[-2.06, -0.23]**	**-2.41**	**.017**	**.043**
		BPFSC (z)	**1.66**	**[0.75, 2.56]**	**3.50**	**.001**	**.002**
		Parent Initiative (z)	0.23	[-0.74, 1.21]	0.46	.645	.774
		Obs Initiative (z)	0.38	[-0.54, 1.30]	0.79	.433	.607
		BPFSC (z) x Parent Initiative (z)	0.30	[-0.70, 1.31]	0.58	.561	.692
		BPFSC (z) x Obs Initiative (z)	**1.42**	**[0.46, 2.37]**	**2.85**	**.005**	**.013**
	Error						
		Residual	3.52	[3.14, 3.96]			
Lability							
	Random						
		Intercept	3.93	[3.12, 4.48]			
	Fixed						
		Intercept	**6.85**	**[5.96, 7.73]**	**14.86**	**.000**	**.000**
		Time	**-1.40**	**[-2.25, -0.55]**	**-3.25**	**.001**	**.004**
		Age	0.62	[-0.35, 1.58]	1.23	.222	.382
		Sex	-0.91	[-1.69, -0.14]	-2.26	.026	.060
		BPFSC (z)	0.57	[-0.22, 1.37]	1.39	.167	.303
		Parent Lability (z)	**3.74**	**[2.91, 4.57]**	**8.66**	**.000**	**.000**
		Obs Lability (z)	0.30	[-0.46, 1.07]	0.76	.448	.607
		BPFSC (z) x Parent Lability (z)	0.71	[-0.04, 1.47]	1.82	.070	.144
		BPFSC (z) x Obs Lability (z)	0.13	[-0.68, 0.93]	0.30	.764	.895
	Error						
		Residual	3.56	[3.16, 4.01]			

*Note*. *Est* Model-provided parameter estimate, *CI* Confidence interval, *t* t-test statistic, *p p-*value, *FDR-p* FDR-corrected *p*-value, *Obs* Observed. Bolded values indicate those that are significant at FDR-corrected *p* < .05. The “(z)” after a variable indicates it is standardized

## Discussion

The current study sought to identify factors that might moderate the association between known preschool-age risk factors and adolescent BPD symptoms and serve as potential targets for early intervention. The moderating effect of observed initiative-taking most closely matched our hypotheses, in that preschool children who had higher levels of internalizing or externalizing psychopathology symptoms and higher levels of observed initiative-taking had fewer BPD symptoms in adolescence than children with higher levels of psychopathology symptoms and lower levels of observed initiative-taking. This finding suggests that higher initiative-taking, which we defined as active intrapersonal and interpersonal coping and resource seeking, may act as a buffer against BPD risk for children placed at higher risk due to their psychopathology symptoms. An unexpected finding, though, was that among children with lower levels of internalizing or externalizing symptoms, higher levels of observed initiative-taking were associated with more BPD symptoms in adolescence. It is notable that these patterns held for both internalizing and externalizing symptoms, and that in both cases, higher initiative-taking was protective only for children who later went on to have symptom scores near or beyond the BPFS-C symptom cut-off point for presumptive BPD diagnosis. While it is reasonable that initiative-taking could help children high in psychopathology symptoms by expanding their active coping repertoire and connecting them to helpful resources, further work should explore why high initiative-taking might add risk for children low in psychopathology symptoms.

Also of interest are our correlational findings, which suggest that our observed measures were distinct from parent-reported measures and are worth exploring in future BPD development research. Our observed measures were at most modestly correlated with parent-reported predictor and moderator measures, indicating that our observed measures likely captured different aspects of temperament and behavior than these other measures. As Stifter et al. [43] summarize, lack of agreement between parent-reported and observed measures does not necessarily indicate that one measure is more accurate than the other, but rather more likely reflects a difference in perspective on the same construct. Our finding that observed measures obtained from short laboratory-based tasks were largely distinct from parent-reported measures of the same construct emphasizes the need for greater use of observational measures and multi-method approaches in BPD development research, as observational measures may identify significant risk, protective, and moderating factors in BPD development that parent/caregiver reports fail to capture.

### Limitations of the current study

Several of our hypotheses were not supported. It is unclear whether this lack of support stems from a genuine lack of moderating effects, or if some of our measures failed to fully capture the underlying constructs. A key limitation of the current study was that our choice of measures for the proposed moderating factors was limited to the assessments conducted at the outset of the Preschool Depression Study, many years prior to the current study, so we were at times unable to choose measures that would be ideally suitable for exploring our hypotheses. For instance, a more relevant parent-reported measure of initiative-taking might include questions about the child’s problem-solving and social-support-seeking behaviors. Additionally, even though we did find a significant moderating effect of observed initiative-taking, an even more relevant measure of observed initiative-taking might assess specific problem-solving behaviors in a task designed to offer multiple opportunities for problem solving, such as a puzzle task where the child could choose to consult an instruction manual or ask for help. Future work would benefit from measures specifically designed to assess our moderating factors, to ensure that nonsignificant results stem from genuine null effects and not from methodological imprecision.

It is also notable that when assessing only individual-level characteristics as moderating factors, there were no significant moderators of the effect of preschool ACEs on adolescent BPD symptoms. As such, it is worth considering whether interpersonal-level or environmental-level factors might have more potential for moderating BPD risk pathways. Future work could examine interpersonal-level and environment-level factors suggested to be moderating factors in BPD development in past retrospective work, such as validating parenting [67], attachment security [68], and positive relationships with non-caregiver adults [21]. Factors found to promote resilience in the context of childhood maltreatment, such as sense of belonging in a community [69] and school engagement [70], may also be worth examining in the context of BPD development.

### Strengths of the current study

Despite the constraints of the measures used, the methodology employed in the current study represents a strength that could inspire new lines of research on BPD development. To our knowledge, there are few other studies that have examined moderating factors in BPD development using prospective, longitudinal data and incorporating both parent reports and laboratory observations of children during preschool. The current study makes a significant contribution to the study of early childhood (preschool-age) precursors of BPD, an area of research that is still largely underdeveloped, as most prior research examining BPD development has done so using school-age or adolescent assessments of risk factors. Our results further underscore the importance of examining patterns of risk at this early age, as well as the potential need for early screening and intervention in children as young as three years old.

An additional strength of the current study is our exploration of initiative-taking, which has rarely been examined in the BPD literature. Adaptive coping strategies and interpersonal skills have been examined in past work on BPD, but not often as part of a unified construct, and not often in early childhood. Initiative-taking stands in contrast to our other potential moderating factors in that it is an adaptive skill that could be enhanced to promote resilience, rather than a maladaptive trait or behavior to be reduced to lessen risk. It is also novel that our observed measure of initiative-taking, which emerged as a significant moderator of risk in two analyses, was comprised of ratings of observed problem-solving, self-starting, and sociable behaviors, rather than self-report ratings of mental processes like cognitive reappraisal and acceptance. The observable nature of our measure facilitates assessment in early childhood, though further work should also explore the mental processes and coping strategies underlying these observed behaviors.

Although not a significant moderator in the current set of analyses, the application of the mean of squared successive differences (MSSD) method (drawn from ecological momentary assessment research) in the coding of cross-sectional observational data to create an observed emotional lability measure is a novel addition to the BPD development literature that could be extended to other studies. This measure allows for the observation of lability of negative affect, not just total negative affect, in young children via behavioral coding of two short, standardized frustration tasks staged in the laboratory, the classroom, or various other settings. Our assessment technique can also transfer to other age groups, given that the MSSD of negative affect can be calculated with observations of other age-appropriate frustration-inducing tasks. Our observed measure may be particularly important given the lack of a correlation between observed and parent-reported emotional lability. Using observed measures of emotional lability may provide a new perspective on lability not often captured in the BPD literature, and should be explored in future work.

### Clinical implications

Our findings that levels of initiative-taking in preschool children significantly affected their risk for later development of BPD points to the need for attention to early risk characteristics and potential interventions in the preschool period. If further work replicates our finding that initiative-taking buffers the effect of high psychopathology symptoms on later BPD symptoms, a critical next step would be developing and testing interventions that foster intrapersonal and interpersonal active coping strategies in early childhood. Some prior work already indicates the importance of active coping for reducing BPD symptoms: for example, patients with BPD who showed improvements in problem-solving over the course of treatment were shown to have better treatment outcomes [71], and the use of positive emotion regulation strategies has been shown to mediate the relationship between better childhood attachment security and lower adolescent BPD symptoms [36]. The latter example also suggests that future work examine whether improvements in active coping can mitigate risk for BPD in the absence of secure attachment with a caregiver.

We focused on moderating factors at the individual level to identify potential targets for intervention that could be implemented in early childhood and in parallel to family-level and social context interventions, or in place of such interventions when family and broader life circumstances are not easily changed. Early interventions promoting initiative-taking and other potential protective factors could be implemented through classroom-based individual-level interventions, some of which have been shown to improve children’s initiative-taking, emotion regulation, and self-control [72–74]. An advantage of classroom-based interventions is their extensive reach, given that most children attend school; when parents are willing and able to be involved, however, other interventions such as Parent–Child Interaction Therapy could also be used to enhance these skills. Skills training and therapy in the early childhood period may have particular efficacy, as the preschool period represents a time of rapid behavioral, social, and neural development during which children may be more responsive to such interventions [75]. Recent work suggests that indicated prevention—taking steps to prevent BPD as soon as features of BPD emerge, instead of waiting for a complete diagnosis—is a critical component of intervention against BPD [76, 77]. Further work will be needed to determine if interventions fostering initiative-taking – including both intrapersonal and interpersonal coping—in young children can indeed significantly mitigate BPD risk conferred by psychopathology.

### Additional future research directions

The current study adds to a limited line of research examining moderating factors in BPD development. Additional work utilizing prospective, longitudinal data and multiple reports will be needed to continue to identify factors in early childhood – as well as middle childhood, late childhood, and adolescence – that moderate the BPD risk pathway. Ideally, future studies will use measures more suited to the moderators being examined; to this end, we suggest that researchers examining developmental trajectories of psychopathology add BPD-related assessments to their longitudinal studies as early as possible. We also suggest that researchers completing the later stages of such longitudinal studies add a BPD symptom assessment to their adolescent or young adult outcome measures, as they might find that more of their sample than predicted endorses a high number of BPD symptoms. Intervention studies assessing the effects of Parent–Child Interaction Therapy, classroom interventions, and other early interventions on psychopathology should also consider adding BPD outcome measures to their work to determine if such interventions might mitigate risk for BPD.

## Conclusions

The current study serves as a starting point for future work on moderating factors in BPD development. Our findings provide evidence for the significant impact of preschool-age initiative-taking on the trajectory of BPD development and imply that fostering active coping and support-seeking in early childhood could help mitigate BPD risk for children high in internalizing or externalizing symptoms. While past work in this field has focused mostly on risk, we take a more strengths-based approach and shift the focus to malleable factors that could be targeted with early intervention to prevent or ameliorate BPD development. Although future work would benefit from more valid measures, our method of using prospective data and multiple reports represents a strength of the current study that should be replicated if possible. In particular, observational measures may provide a more objective report as well as novel targets for screening. Further research is necessary to continue to parse out risk and protective factors in BPD development and to create interventions that might promote resilience and help prevent this debilitating disorder.

## Supplementary Information

**Additional file 1.** R Code for Multilevel Modeling Analyses. This document contains the R code used to perform the 12 multilevel modeling analyses conducted in the current study.

## Data Availability

The datasets used and/or analyzed during the current study are available from the corresponding author on reasonable request.

## Abbreviations

ACEs: Adverse childhood experiences
ADHD: Attention-deficit/ hyperactivity disorder
BPD: Borderline personality disorder
BPFS-C: Borderline Personality Features Scale for Children
DSM: Diagnostic and Statistical Manual of Mental Disorders
HBQ: MacArthur Health and Behavior Questionnaire—Parent Version
Lab-TAB: Preschool Laboratory Temperament Assessment Battery
MSSD: Mean of squared successive differences
PAPA: Preschool Age Psychiatric Assessment
