# Supplementary material for: Identifying moderating factors during the preschool period in the development of borderline personality disorder: a prospective longitudinal analysis
Source: Borderline Personal Disord Emot Dysregul. 2022 Sep 15;9:26. doi: 10.1186/s40479-022-00198-6 (PMC9479250; doi:10.1186/s40479-022-00198-6)
Supplement: Supplementary file 1 — Additional file 1. R Code for Multilevel Modeling Analyses. This document contains the R code used to perform the 12 multilevel modeling analyses conducted in the current study. [file 40479_2022_198_MOESM1_ESM.pdf]

R Code For Multilevel Modeling Analyses

Data

```
bpded_data <- read_sav("~/Desktop/Final_BPD_Sample_Kiran_081122.sav")

bpded_data_short <- bpded_data %>%
  select(Subid, sex, contains("age"), contains("ACES"), contains("EXTL"), contains("INTL"),
         contains("ParentImpulsivity"), contains("ObsImpulsivity"), contains("ParentInitSoc"),
         contains("ObsInitSoc"), contains("ParentLability"), contains("ObsLability"),
         contains("T18BPFSC_T"), contains("T20BPFSC_T"), contains("T18BPD"), contains("T20BPD")) %>%
  select(-EBinterestEngagement, -INSinterestEngagement)
```

Age

```
names(bpded_data_short) = gsub(pattern = "age", replacement = "_age", x = names(bpded_data_short))

bpded_age <- bpded_data_short %>%
  select(Subid, contains("age")) %>%
  pivot_longer(cols = T1_age:T20_age, names_to = "name", values_to = "age")

timepoint <- as.data.frame(unlist(strsplit(bpded_age$name, "_")))
colnames(timepoint) <- "wave"
timepoint <- timepoint %>%
  filter(wave != "age")

bpded_age <- cbind(bpded_age, timepoint)

bpded_age <- bpded_age %>%
  select(Subid, wave, age)
```

ACES

```
bpded_aces <- bpded_data_short %>%
  select(Subid, contains("ACES")) %>%
  select(-contains("Z")) %>%
  pivot_longer(cols = T1_ACES:T20_ACES, names_to = "name", values_to = "ACES")

timepoint <- as.data.frame(unlist(strsplit(bpded_aces$name, "_")))
colnames(timepoint) <- "wave"
timepoint <- timepoint %>%
```

```
  filter(wave != "ACES")

bpded_aces <- cbind(bpded_aces, timepoint)

bpded_aces <- bpded_aces %>%
  select(Subid, wave, ACES)
```

External

```
bpded_extl <- bpded_data_short %>%
  select(Subid, EXTLdim_PS, EXTL_S_T1, EXTL_S_T3) %>%
  pivot_longer(cols = EXTL_S_T1:EXTL_S_T3, names_to = "name", values_to = "EXTL") %>%
  mutate(wave = ifelse(name == "EXTL_S_T1", "T1", ifelse(name == "EXTL_S_T3", "T3", NA))) %>%
  select(Subid, wave, EXTL, EXTLdim_PS)
```

Internal

```
bpded_intl <- bpded_data_short %>%
  select(Subid, INTLdim_PS, INTL_S_T1, INTL_S_T3) %>%
  pivot_longer(cols = INTL_S_T1:INTL_S_T3, names_to = "name", values_to = "INTL") %>%
  mutate(wave = ifelse(name == "INTL_S_T1", "T1", ifelse(name == "INTL_S_T3", "T3", NA))) %>%
  select(Subid, wave, INTL, INTLdim_PS)
```

BPFSC

```
bpded_bpfsc <- bpded_data_short %>%
  select(Subid, contains("BPFSC")) %>%
  pivot_longer(cols = c(T18BPFSC_T, T20BPFSC_T), names_to = "name", values_to = "BPFSC") %>%
  mutate(wave = ifelse(name == "T18BPFSC_T", "T18", ifelse(name == "T20BPFSC_T", "T20", NA))) %>%
  select(-name)
```

Combine All

```
bpd_df <- bpded_age %>%
  full_join(bpded_data_short %>% select(Subid, sex)) %>%
  full_join(bpded_aces) %>%
  full_join(bpded_aces_z) %>%
  full_join(bpded_extl) %>%
  full_join(bpded_intl) %>%
  full_join(bpded_bpfsc) %>%
  group_by(Subid) %>%
  mutate(EXTLdim_PS = max(EXTLdim_PS, na.rm = T),
         INTLdim_PS = max(INTLdim_PS, na.rm = T),
         ZParentImpulsivity = max(ZParentImpulsivity, na.rm = T),
         ZObsImpulsivity = max(ZObsImpulsivity, na.rm = T),
```

```
         ZParentInitSoc = max(ZParentInitSoc, na.rm = T),
         ZObsInitSoc = max(ZObsInitSoc, na.rm = T),
         ZParentLability = max(ZParentLability, na.rm = T),
         BPFSC.max = max(BPFSC, na.rm = T)) %>%
  ungroup()

bpd_df <- do.call(data.frame,
                  lapply(bpd_df,
                         function(x) replace(x, is.infinite(x), NA)))

bpd_df$wave <- gsub("T","",as.character(bpd_df$wave))
bpd_df$wave <- as.numeric(bpd_df$wave)

bpd_df <- bpd_df %>%
  mutate(sex = sex - 1,
         age.T1 = ifelse(wave == 1, age, NA)) %>%
  group_by(Subid) %>%
  mutate(age.T1 = max(age.T1, na.rm = T)) %>%
  ungroup() %>%
  mutate(age.T1.c = age.T1 - 4.499486)

bpfsc <- bpd_df %>%
  select(Subid, BPFSC.max) %>%
  distinct() %>%
  summarize(mean = mean(BPFSC.max),
            sd = sd(BPFSC.max))

obsLab <- bpd_df %>%
  select(Subid, ObsLability) %>%
  distinct() %>%
  summarize(mean = mean(ObsLability, na.rm = T),
            sd = sd(ObsLability, na.rm = T))

bpd_df <- bpd_df %>%
  mutate(wave0 = wave - 1,
         BPFSC.max.z = (BPFSC.max - bpfsc$mean)/bpfsc$sd,
         ZObsLability = (ObsLabilityComp - obsLab$mean)/obsLab$sd) %>%
  group_by(Subid) %>%
  mutate(time = row_number()) %>%
  ungroup() %>%
  mutate(time0 = time - 1) %>%
  relocate(wave0, .after = wave) %>%
  relocate(time, .after = wave0) %>%
  relocate(time0, .after = time) %>%
  mutate(sex.c = ifelse(sex == 1, 1, ifelse(sex == 0, -1, NA))) %>%
  relocate(sex.c, .after = sex)  %>%
  arrange(Subid, wave)
```

Baseline Models

Model 1 (ACES)

```
bmodel_1 <- lmer(ACES ~ 1 + time0 + age.T1.c + sex.c + BPFSC.max.z + (1 | Subid), data = bpd_df,
                 control = lmerControl(optimizer = "bobyqa"))
summary(bmodel_1)
```

Model 2 (INTL)

```
bmodel_2 <- lmer(INTL ~ 1 + time0 + age.T1.c + sex.c + BPFSC.max.z + (1 | Subid), data = bpd_df,
                 control = lmerControl(optimizer = "bobyqa"))
summary(bmodel_2)
```

Model 3 (EXTL)

```
bmodel_3 <- lmer(EXTL ~ 1 + time0 + age.T1.c + sex.c + BPFSC.max.z + (1 | Subid), data = bpd_df,
                 control = lmerControl(optimizer = "bobyqa"))
summary(bmodel_3)
```

Baseline Estimates

Model 1 (ACES)

```
est_1_bm <- tidy(bmodel_1)[c(6:7, 1:5),]
ci_1_bm <- confint.merMod(bmodel_1)
param_1_bm <- cbind(est_1_bm, ci_1_bm) %>%
  mutate(CI = sprintf("[%.2f, %.2f]", `2.5 %`, `97.5 %`)) %>%
  mutate(model = "bmodel_1")
```

Model 2 (INTL)

```
est_2_bm <- tidy(bmodel_2)[c(6:7, 1:5),]
ci_2_bm <- confint.merMod(bmodel_2)
param_2_bm <- cbind(est_2_bm, ci_2_bm) %>%
  mutate(CI = sprintf("[%.2f, %.2f]", `2.5 %`, `97.5 %`)) %>%
  mutate(model = "bmodel_2")
```

Model 3 (EXTL)

```
est_3_bm <- tidy(bmodel_3)[c(6:7, 1:5),]
ci_3_bm <- confint.merMod(bmodel_3)
param_3_bm <- cbind(est_3_bm, ci_3_bm) %>%
  mutate(CI = sprintf("[%.2f, %.2f]", `2.5 %`, `97.5 %`)) %>%
  mutate(model = "bmodel_3")

bmodel_params <- rbind(param_1_bm, param_2_bm, param_3_bm)
```

Moderator Models

Model 1 (ACES)

```
# impulsivity
model_1_imp <- lmer(ACES ~ 1 + time0 + age.T1.c + sex.c + BPFSC.max.z +
                      ZParentImpulsivity + ZObsImpulsivity +
                      BPFSC.max.z:ZParentImpulsivity + BPFSC.max.z:ZObsImpulsivity +
                      (1 | Subid), data = bpd_df, control = lmerControl(optimizer = "bobyqa"))
summary(model_1_imp)

# init soc
model_1_initsoc <- lmer(ACES ~ 1 + time0 + age.T1.c + sex.c + BPFSC.max.z +
                          ZParentInitSoc + ZObsInitSoc +
                          BPFSC.max.z:ZParentInitSoc + BPFSC.max.z:ZObsInitSoc +
                          (1 | Subid), data = bpd_df, control = lmerControl(optimizer = "bobyqa"))
summary(model_1_initsoc)

# lability
model_1_lability <- lmer(ACES ~ 1 + time0 + age.T1.c + sex.c + BPFSC.max.z +
                           ZParentLability + ZObsLability +
                           BPFSC.max.z:ZParentLability + BPFSC.max.z:ZObsLability +
                           (1 | Subid), data = bpd_df, control = lmerControl(optimizer = "bobyqa"))
summary(model_1_lability)
```

Model 2 (INTL)

```
# impulsivity
model_2_imp <- lmer(INTL ~ 1 + time0 + age.T1.c + sex.c + BPFSC.max.z +
                      ZParentImpulsivity + ZObsImpulsivity +
                      BPFSC.max.z:ZParentImpulsivity + BPFSC.max.z:ZObsImpulsivity +
                      (1 | Subid), data = bpd_df, control = lmerControl(optimizer = "bobyqa"))
summary(model_2_imp)

# init soc
model_2_initsoc <- lmer(INTL ~ 1 + time0 + age.T1.c + sex.c + BPFSC.max.z +
                          ZParentInitSoc + ZObsInitSoc +
                          BPFSC.max.z:ZParentInitSoc + BPFSC.max.z:ZObsInitSoc +
                          (1 | Subid), data = bpd_df, control = lmerControl(optimizer = "bobyqa"))
summary(model_2_initsoc)
```

```
# lability
model_2_lability <- lmer(INTL ~ 1 + time0 + age.T1.c + sex.c + BPFSC.max.z +
                           ZParentLability + ZObsLability +
                           BPFSC.max.z:ZParentLability + BPFSC.max.z:ZObsLability +
                           (1 | Subid), data = bpd_df, control = lmerControl(optimizer = "bobyqa"))
summary(model_2_lability)
```

Model 3 (EXTL)

```
# impulsivity
model_3_imp <- lmer(EXTL ~ 1 + time0 + age.T1.c + sex.c + BPFSC.max.z +
                      ZParentImpulsivity + ZObsImpulsivity +
                      BPFSC.max.z:ZParentImpulsivity + BPFSC.max.z:ZObsImpulsivity +
                      (1 | Subid), data = bpd_df, control = lmerControl(optimizer = "bobyqa"))
summary(model_3_imp)

# init soc
model_3_initsoc <- lmer(EXTL ~ 1 + time0 + age.T1.c + sex.c + BPFSC.max.z +
                          ZParentInitSoc + ZObsInitSoc +
                          BPFSC.max.z:ZParentInitSoc + BPFSC.max.z:ZObsInitSoc +
                          (1 | Subid), data = bpd_df, control = lmerControl(optimizer = "bobyqa"))
summary(model_3_initsoc)

# lability
model_3_lability <- lmer(EXTL ~ 1 + time0 + age.T1.c + sex.c + BPFSC.max.z +
                           ZParentLability + ZObsLability +
                           BPFSC.max.z:ZParentLability + BPFSC.max.z:ZObsLability +
                           (1 | Subid), data = bpd_df, control = lmerControl(optimizer = "bobyqa"))
summary(model_3_lability)
```

Moderator Estimates

Model 1 (ACES)

```
est_1_imp <- tidy(model_1_imp)[c(10:11,1:9),]
ci_1_imp <- confint.merMod(model_1_imp)
param_1_imp <- cbind(est_1_imp, ci_1_imp) %>%
  mutate(CI = sprintf("[%.2f, %.2f]", `2.5 %`, `97.5 %`)) %>%
  mutate(model = "model_1_imp")

est_1_initsoc <- tidy(model_1_initsoc)[c(10:11,1:9),]
ci_1_initsoc <- confint.merMod(model_1_initsoc)
param_1_initsoc <- cbind(est_1_initsoc, ci_1_initsoc) %>%
  mutate(CI = sprintf("[%.2f, %.2f]", `2.5 %`, `97.5 %`)) %>%
  mutate(model = "model_1_initsoc")

est_1_lability <- tidy(model_1_lability)[c(10:11,1:9),]
ci_1_lability <- confint.merMod(model_1_lability)
```

```r
param_1_lability <- cbind(est_1_lability, ci_1_lability) %>%
  mutate(CI = sprintf("[%.2f, %.2f]", `2.5 %`, `97.5 %`)) %>%
  mutate(model = "model_1_lability")

param_1_mod <- rbind(param_1_imp, param_1_initsoc, param_1_lability)
```

Model 2 (INTL)

```r
est_2_imp <- tidy(model_2_imp)[c(10:11,1:9),]
ci_2_imp <- confint.merMod(model_2_imp)
param_2_imp <- cbind(est_2_imp, ci_2_imp) %>%
  mutate(CI = sprintf("[%.2f, %.2f]", `2.5 %`, `97.5 %`)) %>%
  mutate(model = "model_2_imp")

est_2_initsoc <- tidy(model_2_initsoc)[c(10:11,1:9),]
ci_2_initsoc <- confint.merMod(model_2_initsoc)
param_2_initsoc <- cbind(est_2_initsoc, ci_2_initsoc) %>%
  mutate(CI = sprintf("[%.2f, %.2f]", `2.5 %`, `97.5 %`)) %>%
  mutate(model = "model_2_initsoc")

est_2_lability <- tidy(model_2_lability)[c(10:11,1:9),]
ci_2_lability <- confint.merMod(model_2_lability)
param_2_lability <- cbind(est_2_lability, ci_2_lability) %>%
  mutate(CI = sprintf("[%.2f, %.2f]", `2.5 %`, `97.5 %`)) %>%
  mutate(model = "model_2_lability")

param_2_mod <- rbind(param_2_imp, param_2_initsoc, param_2_lability)
```

Model 3 (EXTL)

```r
est_3_imp <- tidy(model_3_imp)[c(10:11,1:9),]
ci_3_imp <- confint.merMod(model_3_imp)
param_3_imp <- cbind(est_3_imp, ci_3_imp) %>%
  mutate(CI = sprintf("[%.2f, %.2f]", `2.5 %`, `97.5 %`)) %>%
  mutate(model = "model_3_imp")

est_3_initsoc <- tidy(model_3_initsoc)[c(10:11,1:9),]
ci_3_initsoc <- confint.merMod(model_3_initsoc)
param_3_initsoc <- cbind(est_3_initsoc, ci_3_initsoc) %>%
  mutate(CI = sprintf("[%.2f, %.2f]", `2.5 %`, `97.5 %`)) %>%
  mutate(model = "model_3_initsoc")

est_3_lability <- tidy(model_3_lability)[c(10:11,1:9),]
ci_3_lability <- confint.merMod(model_3_lability)
param_3_lability <- cbind(est_3_lability, ci_3_lability) %>%
  mutate(CI = sprintf("[%.2f, %.2f]", `2.5 %`, `97.5 %`)) %>%
  mutate(model = "model_3_lability")

param_3_mod <- rbind(param_3_imp, param_3_initsoc, param_3_lability)
```

FDR Correction

```
param_mod <- rbind(param_1_mod, param_2_mod, param_3_mod)

fdr <- rbind(bmodel_params, param_mod)

fdr <- fdr %>%
  mutate(new_p = round(p.adjust(fdr$p.value, "BH"), 3))
```
